# Differential Impact of eHealth Literacy on Wellness Behaviors of Iranian Nurses: Descriptive Correlational Cross-Sectional Study

**DOI:** 10.2196/80792

**Published:** 2025-10-09

**Authors:** Behnam Moradi, Mohammad Javad Hosseinabadi-Farahani, Mohammadreza Dinmohammadi, Mohammad Saatchi

**Affiliations:** 1 Department of Nursing, School of Behavior Sciences, University of Social Welfare and Rehabilitation Sciences, Velenjak, Daneshjo Blvd, Kadayar Dead End, Tehran, 1985713871, Iran, +98 9192158179; 2 Social Determinants of Health Research Center, University of Social Welfare and Rehabilitation Sciences, Tehran, Iran; 3 Health in Emergency and Disaster Research Center, University of Social Welfare and Rehabilitation Sciences, Tehran, Iran; 4 Department of Biostatistics and Epidemiology, University of Social Welfare and Rehabilitation Sciences, Tehran, Iran

**Keywords:** eHealth literacy, healthy lifestyle, nurses, health-promoting behaviors, digital health

## Abstract

**Background:**

Nurses play a pivotal role in health care delivery and health education. However, their demanding work environments, characterized by irregular shifts and high stress, often hinder their ability to adopt healthy lifestyles, compromising both their well-being and their effectiveness as role models for health promotion. With the rise of digital health technologies, eHealth literacy—the capacity to seek, evaluate, and apply online health information—has emerged as a critical factor influencing health-promoting behaviors among health care professionals.

**Objective:**

This study aims to examine the association between eHealth literacy and healthy lifestyle behaviors among Iranian nurses, focusing on nutrition, physical activity, stress management, health responsibility, interpersonal relations, and spiritual growth.

**Methods:**

We conducted a cross-sectional descriptive-analytical study in Tehran, Iran, from November 2024 to February 2025. A total of 334 registered nurses from 7 public and teaching hospitals participated. Data were collected via the eHealth Literacy Scale and the Health-Promoting Lifestyle Profile II. Spearman correlation and multivariate linear regression analyses were performed, with statistical significance set at *P*<.05.

**Results:**

Of 334 nurses, 234 (70.1%) had moderate eHealth literacy, 178 (53.3%) had good healthy lifestyle scores, and none scored low. A significant positive correlation was found between eHealth literacy and overall healthy lifestyle (*r*=0.565; *P*<.001), with the strongest associations observed for spiritual growth (*r*=0.537), health responsibility (*r*=0.437), and interpersonal relationships (*r*=0.467). Associations with stress management (*r*=0.318), nutrition (*r*=0.321), and physical activity (*r*=0.289) were weaker but remained statistically substantial.

**Conclusions:**

Higher eHealth literacy is associated with healthier lifestyles, particularly in the areas of spiritual growth and health responsibility. Workplace barriers, such as rotating shifts, limit physical activity and stress management. Targeted eHealth training and wellness programs are needed.

## Introduction

### Background

In recent decades, the global shift in disease patterns has led health care systems to transition their focus from treatment to prevention and health promotion [1]. In 2016, it was estimated that 40.5 million (71%) of the 56.9 million deaths worldwide were due to noncommunicable diseases (NCDs), and projections suggest that by 2030, these diseases will account for more than 75% of the global mortality [2-4]. Research indicates that the majority of NCDs are rooted in unhealthy behaviors and lifestyle factors, including poor nutrition, physical inactivity, chronic stress, and tobacco use [5-8]. Nurses, as the cornerstone of health care systems, play a pivotal role in promoting preventive behaviors to combat NCDs, yet their own lifestyle practices warrant closer examination due to their unique occupational challenges [9,10].

According to the World Health Organization, a healthy lifestyle is defined as a set of everyday behaviors and routines that, by reducing risk factors, promote physical, mental, and social well-being and improve quality of life [11]. In this context, nurses—who constitute the largest caregiving group within Iran’s health care system and play a vital role in both care delivery and health education—are simultaneously considered health services providers and role models for the community [12,13]. However, owing to irregular work shifts, persistent occupational stress, and physical and emotional exhaustion, nurses are especially vulnerable to the negative outcomes of unhealthy lifestyles [14,15].

Although nurses play an active role in educating patients about healthy lifestyle practices [13], research indicates that a considerable number of nurses face challenges in sustaining health-promoting behaviors within their personal lives [16,17]. For example, a study by Priano et al [18] reported that fewer than 5% of nurses adhered to a healthy lifestyle, which includes components such as proper diet, regular physical activity, maintaining a healthy weight, and abstaining from smoking. The same study revealed that 72% of nurses did not engage in physical activity, and 61% reported poor dietary patterns. Such deficiencies may adversely affect the quality of care and nurses’ professional functioning, as unhealthy lifestyle behaviors have been associated with increased job stress, higher levels of burnout, and reduced quality of working life, which can in turn compromise patient care and caring behaviors [19-21].

While previous research has examined a variety of individual, social, and environmental factors affecting nurses’ lifestyles, the emergence of eHealth literacy has recently drawn attention as a potential determinant of health-promoting behaviors [22]. eHealth literacy refers to individuals’ ability to search, evaluate, and apply health information obtained from digital environments [23,24], and it plays a vital role in self-management, informed decision-making, and preventive behavior engagement [25,26].

Recent studies have revealed significant associations between eHealth literacy and various dimensions of a healthy lifestyle, including nutrition, physical activity, and stress management [25,27-29]. For example, research conducted among adult internet users in Japan indicated that certain health behaviors, such as physical activity and balanced nutrition, were independently associated with higher levels of eHealth literacy [30]. In contrast, a study among hospital nurses in South Korea revealed weak relationships between eHealth literacy and several lifestyle dimensions, such as exercise and diet, suggesting a gap between health knowledge and actual behavior [27]. These heterogeneous findings highlight the need for further investigation.

Moreover, while studies in Western and East Asian contexts have explored eHealth literacy among nurses [25,27,31], limited research has addressed this topic in the Middle Eastern context, particularly among Iranian nurses, where cultural and professional factors may uniquely influence health behaviors [32]. Given the unique sociocultural and occupational challenges faced by Iranian nurses, such as high workloads [33,34] and limited access to digital health resources [35], investigating eHealth literacy in this population is critical for developing targeted interventions.

Considering the critical importance of healthy lifestyle behaviors for nurses’ health and professional functioning [17] and the increasing role of digital health technologies in modern health care delivery [36], it is important to examine the interaction of these factors.

### Objectives

This study aimed to investigate the association between eHealth literacy and healthy lifestyle behaviors among Iranian nurses, focusing on specific dimensions such as nutrition, physical activity, stress management, health responsibility, interpersonal relations, and spiritual growth.

## Methods

### Ethical Considerations

This study was conducted in accordance with the Declaration of Helsinki [37] and approved by the Ethics Committee of the University of Social Welfare and Rehabilitation Sciences (IR.USWR.REC.1403.174). All participants provided written informed consent after receiving comprehensive information about the study’s purpose, confidentiality protocols, and their right to withdraw at any time without consequences. To ensure privacy and confidentiality, the questionnaires were completed anonymously without collecting names or personal identifiers. Each questionnaire was assigned a code number, and the completed forms were stored in a locked cabinet accessible only to the research team. Electronic data were kept on a password-protected computer. No compensation or monetary incentives were provided to participants. The study posed no significant risks and offered potential benefits through insights for improving nursing services and patient satisfaction.

### Study Design, Setting, and Participants

This cross-sectional descriptive-analytical study was in Tehran, Iran, over a 4-month period from November 2024 to February 2025. The target population consisted of all 10,971 registered nurses [38] working in public and teaching hospitals affiliated with the Ministry of Health and Medical Education, including those under the supervision of Tehran University of Medical Sciences, Iran University of Medical Sciences, Shahid Beheshti University of Medical Sciences, and the University of Social Welfare and Rehabilitation Sciences.

The eligibility criteria included holding a bachelor’s degree in nursing, having at least 1 year of clinical experience, being employed in any formal capacity (permanent, contractual, agreement-based, or project-based), and not having any physical limitations that would impair job performance. Participants who submitted incomplete responses were excluded from the study.

### Sampling

**Figure 1.**
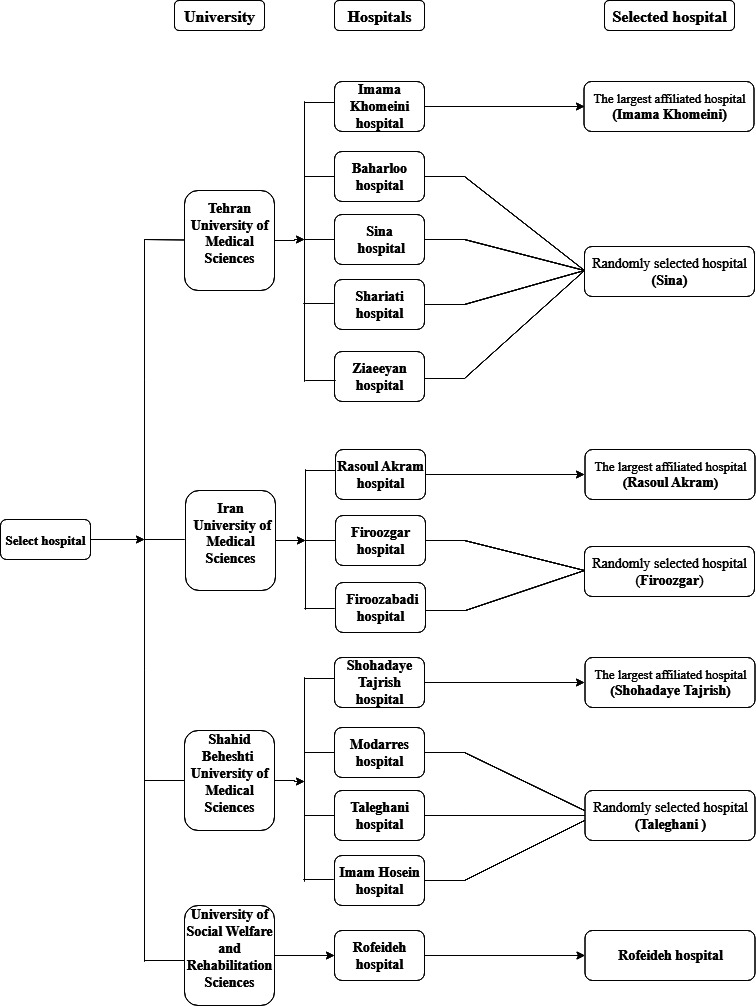
Selection of the hospital flowchart.

A flowchart illustrating the participant selection process is provided in Multimedia Appendix 1. The sampling strategy followed a multistage approach. Tehran’s 4 main universities cover distinct geographic areas of the city: Shahid Beheshti (north, northeast, and east), Tehran (center and south), Iran (west and northwest), and the University of Social Welfare and Rehabilitation Sciences (southeast). On the basis of this regional division, 2 general hospitals from each university were selected. Hospital selection was conducted via cluster random sampling, with one being the largest general teaching hospital and the other chosen randomly from the remaining eligible hospitals through a simple lottery. Single-specialty hospitals were excluded from the sample, and in total, 8 hospitals were included (Figure 1).

The sample size was calculated on the basis of parameters derived from the study by Cho et al [27], which investigated associations between eHealth literacy and health-promoting behaviors among hospital nurses in South Korea. A correlation coefficient of 0.2 was used as the estimated effect size, corresponding to a small-to-moderate association based on the Cohen criteria [39]. This value was selected because Cho et al [27] reported a standardized regression coefficient (β approximately 0.20) for the association between eHealth literacy and overall health-promoting behavior, which aligns with our target analysis. Assuming a power of 80%, an α of .05, and a design effect of 1.2 to account for cluster sampling, the minimum required sample size was calculated to be 334 via STATA software (StataCorp LLC).

On the basis of the estimated sample size (N=334), the plan was to recruit at least 100 nurses from each of the 3 main universities—Tehran University of Medical Sciences, Iran University of Medical Sciences, and Shahid Beheshti University of Medical Sciences. The remaining participants were drawn from a single eligible hospital affiliated with the University of Social Welfare and Rehabilitation Sciences. Nurses at each hospital were selected via convenience sampling, considering their work shifts and availability. While the goal was to enroll approximately 50 nurses per selected hospital, the actual number of nurses varied slightly due to sampling constraints.

### Data Collection Process

Questionnaires were distributed by the researcher during shift breaks or staff meetings at the selected hospitals. More questionnaires were distributed than the final number of participants to account for potential nonresponses. Nurses were invited to participate voluntarily after providing informed consent. To minimize missing data, the researcher immediately reviewed each completed questionnaire and asked participants to clarify any unanswered or ambiguous items. This procedure yielded 334 fully completed questionnaires for analysis.

### Instruments

The instruments used in this study consisted of 3 main sections: (1) demographic Questionnaire, (2) eHealth Literacy Scale (eHEALS), and (3) Health-Promoting Lifestyle Profile II (HPLP-II).

#### Demographic Questionnaire

This section gathered information on participants’ age (categorized as 20‐29, 30‐39, 40‐49, and ≥50 y), gender (man and woman), marital status (married and unmarried), education level (bachelor’s, master’s, and PhD), work experience (1‐5, 5‐10, 10‐15, and 15‐20 y), work shift (morning, evening, night, and rotating), and affiliated university (Tehran University of Medical Sciences, Iran University of Medical Sciences, Shahid Beheshti University of Medical Sciences, University of Social Welfare and Rehabilitation Sciences).

#### eHealth Literacy Scale

eHEALS is a standardized tool that includes 8 items rated on a Likert scale from 1=very low to 5=very high, producing a total score ranging from 8 to 40 [23]. In a cross-sectional study by Bazm et al [40], the Persian version of the eHEALS was validated among 525 young individuals in Yazd, Iran, yielding a Cronbach α of 0.933. Scores are interpreted as follows: low (8-22), moderate (23-32), and high (33-40) [23,33-36,38-42] [23,33-36,38-42].

#### Health-Promoting Lifestyle Profile II

HPLP-II scale comprises 49 items across 6 subdomains: nutrition (dietary habits and food choices), physical activity (engagement in regular exercise), health responsibility, stress management (recognizing stressors and applying coping strategies), interpersonal relations (fostering close and supportive relationships), and spiritual growth or self-actualization (developing purpose, self-awareness, and personal growth). Responses are rated on a 4-point Likert scale: 1=never, 2=sometimes, 3=often, and 4=usually. Subscale scores are interrelated and collectively contribute to a total score, which ranges from 49 to 196. Total scores were categorized as low (49-98), moderate (99-147), good (148-171), and excellent (172-196) [42]. The questionnaire’s validity and reliability were supported by a study conducted by Mohammadi Zeidi et al [43], reporting a Cronbach α of 0.82.

### Data Analysis

The data obtained from the questionnaires were statistically analyzed via SPSS software (version 24; IBM Corp) in 3 sections: descriptive statistics (frequencies, percentages, means, and SDs) were calculated for demographic variables (age, gender, marital status, education, work experience, work shift, and affiliated university), eHealth literacy (eHEALS scores), and healthy lifestyle (HPLP-II scores and subscales). The Kolmogorov-Smirnov test assessed normality for eHealth literacy and healthy lifestyle scores. Due to the nonnormal distribution of eHealth literacy scores (*P*<.001), the Spearman correlation was used to examine associations between eHealth literacy and healthy lifestyle subscales (nutrition, physical activity, health responsibility, stress management, interpersonal relations, and spiritual growth).

To compare demographic characteristics across eHealth literacy and healthy lifestyle scores, Mann-Whitney *U* tests were used for binary variables (eg, gender and marital status), and Kruskal-Wallis tests were applied for categorical variables with more than 2 levels (eg, age groups, education level, work experience, and work shift).

Multivariate linear regression with backward elimination identified confounders (eHealth literacy: age, work experience, and work shift; healthy lifestyle: work experience and work shift). Linear regression was then used to assess the association between eHealth literacy and healthy lifestyle scores, while controlling for these confounders. A *P* value of<.05 was considered to indicate statistical significance.

## Results

### Demographic Characteristics of the Study

The results indicated that the majority of participants were aged 20 to 29 years. The gender distribution was nearly equal, with most participants being single. In terms of educational attainment, the largest proportion held a bachelor’s degree, while the most common work experience ranged from 5 to 10 years. With respect to work shifts, 55.9% (187/334) of the participants worked rotating shifts (Table 1).

**Table 1.** Frequency distributions and percentages of demographic variables among nurse participants (N=334).

Demographic variables	Participants, n (%)	
Age (y)		
20‐29	195 (58.4)	
30‐39	100 (29.9)	
40‐49	39 (11.7)	
≥50	0 (0)	
Gender		
Man	148 (44.3)	
Woman	186 (55.7)	
Marital status		
Married	114 (34.1)	
Unmarried	220 (65.9)	
Work shift		
Morning	47 (14.1)	
Evening	16 (4.8)	
Night	84 (25.1)	
Rotating	187 (56)	
Education level		
Bachelor’s degree	286 (85.6)	
Master’s degree	45 (13.5)	
PhD	3 (0.9)	
Work experience (y)		
1-5	95 (28.4)	
5-10	124 (37.1)	
10-15	92 (27.5)	
15-20	23 (6.9)	
≥20	0 (0)	
Affiliated university		
Iranian Medical Sciences	120 (35.9)	
Tehran Medical Sciences	91 (27.2)	
Shahid Beheshti Medical Sciences	96 (28.7)	
Social Welfare and Rehabilitation Sciences	27 (8.1)	

### eHealth Literacy and Healthy Lifestyle Outcomes

The overall scores for eHealth literacy and healthy lifestyle among nurses were moderate and good, respectively. The eHealth literacy levels were distributed as follows: 19.4% (65/334) nurses had poor levels, 70.1% (234/334) nurses had moderate levels, and 10.5% (35/334) nurses had strong levels. With respect to healthy lifestyle scores, 44.6% (149/334) nurses were at moderate levels, 53.3% (178/334) nurses were at good levels, and 2.1% (7/334) nurses were at excellent levels. No nurses in this study were found to have low healthy lifestyle scores.

The Kolmogorov-Smirnov test was used to check univariate normality, and only healthy lifestyle scores were normally distributed (*P*=.20). In contrast, eHealth literacy scores were nonnormally distributed (*P*<.001).

**Figure 2.**
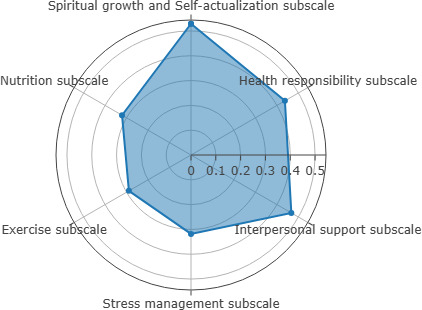
eHealth literacy and healthy lifestyle dimension.

The results revealed a significant positive correlation between eHealth literacy and healthy lifestyles among nurses, as determined by the Spearman correlation coefficient (Table 2 and Figure 2).

**Table 2.** Spearman correlation coefficients between eHealth literacy and healthy lifestyle among nurses (N=334)^a^.

Variables	eHealth literacy	
	*r*	*P* value
Healthy lifestyle	0.565	<.001
Spiritual growth and self-actualization subscale	0.529	<.001
Health responsibility subscale	0.437	<.001
Interpersonal support subscale	0.467	<.001
Stress management subscale	0.318	<.001
Exercise subscale	0.289	<.001
Nutrition subscale	0.321	<.001

a *P*≤.05 was considered statistically significant.

Multivariate linear regression analysis was used to identify factors influencing nurses’ eHealth literacy and healthy lifestyles. The initial model included age, gender, education level, work shift, and work experience. With backward elimination, nonsignificant variables were progressively removed. The final eHealth literacy model identified age group, work experience, and work shift as the most influential factors. For healthy lifestyles, however, only work experience and work shifts were significant predictors (Table 3).

The results of the ANOVA indicated that the overall regression model predicting a healthy lifestyle based on eHealth literacy was statistically significant (*F*_3,3_=86.030; *P*<.001), suggesting a good model fit. The mean square for the regression source was 1110.673, whereas the mean square for the residual was 12.810. This confirms that the model significantly explains a substantial portion of the variance in healthy lifestyle scores among nurses.

As shown in Table 4, the results of the multiple linear regression analysis indicated that eHealth literacy was a statistically significant predictor of healthy lifestyle scores among nurses, even when controlling for work shift and experience variables.

**Table 3.** Multiple linear regressions to determine confounders between the main and dependent variables^a^.

Variables	Coefficient (SE)	*P* value
eHealth literacy		
Age (y)		
30-39	−0.41 (0.84)	.63
40-49	−7.55 (1.28)	<.001
Work experience (y)		
5‐10	−0.34 (0.66)	.61
10-15	2.46 (1.08)	.02
15‐20	1.66 (1.56)	.29
Work shift		
Evening	−8.19 (1.55)	<.001
Night	−3.39 (0.95)	<.001
Rotating	−3.02 (0.86)	0.001
Healthy lifestyle		
Work experience (y)		
5‐10	−4.86 (2.04)	.02
10‐15	1.31 (2.19)	.55
15‐20	−2.01 (4.18)	.63
Work shift		
Evening	−16.42 (4.37)	<.001
Night	−2.46 (2.98)	.41
Rotating	−3.36 (2.81)	.23

a *P*≤.05 was considered statistically significant.

**Table 4.** Multiple linear regression coefficients for predicting healthy lifestyles on the basis of eHealth literacy, controlling for work experience and shift type.

Variable	*P* value	*t* test (*df*)	Standardized coefficient β (SE)		Unstandardized coefficient (B)
Constant	<.001	14.208 (333)	—^a^ (4.901)		69.635
eHealth literacy	<.001	15.914 (333)	0.663 (0.132)		2.096
Work experience	.04	–1.791 (333)	–0.355 (0.234)		–0.558
Shift type	.05	–1.677 (333)	–0.300 (0.254)		–0.748

a The empty cell in "Constant" indicates that β is not calculated for the regression intercept.

## Discussion

### Principal Findings

This study investigated the association between eHealth literacy and multiple dimensions of a healthy lifestyle among Iranian nurses. The findings revealed a statistically significant and positive correlation between eHealth literacy and overall healthy lifestyle scores. Notably, the strongest associations were observed in the domains of spiritual growth, health responsibility, and interpersonal relationships, whereas weaker correlations were found in physical activity and stress management.

The high prevalence of moderate eHealth literacy (234/334, 70.1%) and healthy lifestyles (178/334, 53.3%) among participants suggests that while Iranian nurses possess a reasonable baseline of eHealth literacy and wellness behaviors, there remains substantial room for improvement. The positive association identified in this study is consistent with prior research, including studies by Cho et al [27], Gartrell et al [25], and Yogurtcu et al [44], which reported that higher eHealth literacy levels were linked to more frequent engagement in health-promoting behaviors such as exercise, healthy eating, and stress reduction.

Among the different lifestyle dimensions, spiritual growth emerged as the dimension most strongly linked to eHealth literacy. This suggests that nurses who are more digitally literate may be more inclined to engage in self-reflection, pursue personal development, and find purpose in life. Such tendencies are often supported by digital access to motivational content, mindfulness resources, and health-oriented communities. In the Iranian sociocultural context, where spirituality plays an important role in both personal and professional life [45,46], this relationship may be especially pronounced. Health responsibility was also notably associated with eHealth literacy. Nurses with stronger digital skills, such as seeking reliable online health information, monitoring personal health indicators, and engaging in preventive actions, are more likely to take an active role in managing their own health. This finding aligns with previous studies by Cho et al [27], Wilandika et al [47], and Yogurtcu et al [44], which emphasized the role of digital health literacy in facilitating self-care and proactive health behavior.

The association between eHealth literacy and interpersonal relationships suggests that digital competencies may enhance nurses’ ability to sustain meaningful personal and professional connections. Such skills can support emotional resilience and social cohesion, particularly in high-stress clinical settings such as hospital wards. Maintaining strong social relationships is vital for nurses’ psychological well-being and overall quality of life. These findings are in line with the studies conducted by Cho et al [27] and Gartrell et al [25], which indicated that higher levels of eHealth literacy among nurses are significantly linked to better interpersonal relationships and improved nursing performance.

In contrast, the association between eHealth literacy and the dimensions of stress management, physical activity, and nutrition was less prominent. These results indicate that possessing the knowledge and skills to access digital health information does not automatically translate into effective behavioral change in all areas. For stress management, even well-informed nurses may struggle to implement coping strategies due to demanding workloads, emotional fatigue, and limited institutional support. Likewise, physical activity and healthy eating habits may be hindered by long shifts, time constraints, or a lack of access to nutritious food and exercise facilities within the workplace. These observations are consistent with the findings of Cho et al [27] and Yogurtcu et al [44], who reported similar patterns in their studies.

These findings underscore the need for multidimensional interventions that enhance eHealth literacy while addressing workplace barriers. For instance, tailored eHealth training programs can improve nurses’ ability to access reliable digital health resources [25,31,48]. In addition, flexible scheduling and on-site wellness facilities (eg, exercise spaces or nutrition counseling) can mitigate barriers such as rotating shifts, enabling nurses to adopt healthier behaviors [17,49,50].

### Strengths and Limitations

This study used self-administered questionnaires to collect data, with participants encouraged to provide responses as accurately and honestly as possible. Nonetheless, this approach is subject to potential biases stemming from factors such as participants’ emotional states or inherent cognitive distortions. However, immediate review of responses by research assistants minimized missing data. The convenience sampling approach limits generalizability to broader nursing populations, but the inclusion of nurses from multiple hospitals in Tehran enhances representativeness within this context. Furthermore, the cross-sectional nature of the study design limits the ability to infer causal associations among the examined variables; future longitudinal studies could address this.

Despite these limitations, the study’s strengths include a robust sample size (N=334), the use of validated instruments (eHEALS and HPLP-II), and a comprehensive analysis of multiple lifestyle dimensions, providing valuable insights into eHealth literacy among Iranian nurses.

### Conclusions

This study highlights the important role of eHealth literacy in promoting healthy lifestyle behaviors among nurses. While certain dimensions, such as spiritual growth and health responsibility, appear to be more strongly influenced by digital competence, others require additional institutional and environmental support to translate awareness into sustained action. Strengthening eHealth literacy should therefore be viewed not as a stand-alone solution but as one element within a comprehensive strategy to enhance the health and well-being of the nursing workforce.

## Supplementary material

10.2196/80792 Multimedia Appendix 1 This flowchart illustrates the study process, starting with a statistical population of 109,711 nurses. It outlines the population assessment, inclusion/exclusion criteria (eg, at least 1 year of nursing degree, no work-limiting illness), and sample size determination (n=334) based on Cho et al [27] and STATA software. The sampling method, involving 8 hospitals and 4 medical universities, and the analysis using SPSS v24 for descriptive, Spearman correlation, and linear regression tests are also depicted.

## Abbreviations

eHEALS: eHealth Literacy Scale
HPLP-II: Health-Promoting Lifestyle Profile II
NCD: noncommunicable disease
