# Supplementary figures and images for: Differential Impact of eHealth Literacy on Wellness Behaviors of Iranian Nurses: Descriptive Correlational Cross-Sectional Study
Source: Asian Pac Isl Nurs J. 2025 Oct 9;9:e80792. doi: 10.2196/80792 (PMC12605288; doi:10.2196/80792)

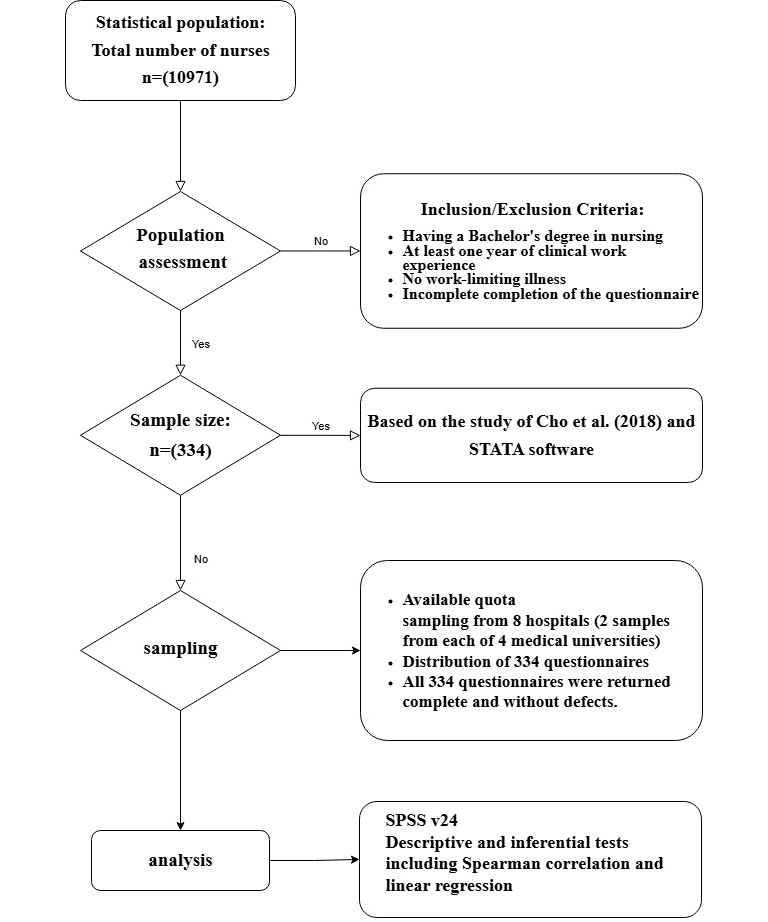

Supplement: Multimedia Appendix 1 [file apinj-v9-e80792-s001.png]
